# Adsorption characteristics and mechanisms of Cd^2+^ from aqueous solution by biochar derived from corn stover

**DOI:** 10.1038/s41598-022-22714-y

**Published:** 2022-10-21

**Authors:** Fang Chen, Yaosheng Sun, Chao Liang, Tianyu Yang, Shican Mi, Yehong Dai, Molin Yu, Qiang Yao

**Affiliations:** 1 School of Resources and Civil Engineering, Northeastern University, Shenyang, 110819 China; 2 School of Resources and Materials, Northeastern University at Qinhuangdao, Qinhuangdao, 066004 China; 3 Ocean College, Hebei Agriculture University, Qinhuangdao, 066004 China

**Keywords:** Ecology, Environmental sciences

## Abstract

Corn stover could be pyrolysed to prepare biochar for removing pollutants in water and realizing the resource utilization of biomass. The aims of the present study were to investigate the optimal preparation and adsorption conditions of biochar and to reveal the adsorption characteristics and mechanisms of Cd^2+^ in water by biochar. For this purpose, with Cd^2+^ as the target pollutant, the pyrolysis conditions involved in the pyrolysis temperature, retention time, and heating rate were evaluated and optimized. Additionally, the characteristics, mechanisms and optimal adsorption conditions of Cd^2+^ by biochar were determined. A series of characterization techniques was employed, including scanning electron microscopy (SEM), Fourier transform infrared spectroscopy (FTIR), X-ray diffraction (XRD) and specific surface area analysis (S_BET_). The optimum pyrolysis parameters were a pyrolysis temperature of 700 °C, a retention time of 2.5 h, and a heating rate of 5 °C/min. Acid/base modification did not improve the adsorption capacity of biochar. The Langmuir and the Elovich model were the most suitable isotherm and kinetic models for equilibrium data, respectively. The maximum adsorption capacity fitted by Langmuir model was 13.4 mg/g. Furthermore, mineral precipitation and π electron interactions were shown to be the main adsorption mechanisms of Cd^2+^. The optimum adsorption conditions for Cd^2+^ in water were a CaCl_2_ electrolyte solution of 0.01 mol/L, a pH level of 6.7, and a biochar dosage of 0.4 g. Our results indicated that corn stover biochar was an appropriate approach for improving the status of water with Cd^2+^ contamination in the short term and for promoting a new perspective for the rational utilization of corn stover and the low-cost pollution control of heavy metals in water.

## Introduction

Cadmium (Cd^2+^) is a harmful heavy metal element that is ubiquitous in aquatic environments^1,2^. Cd^2+^ has the characteristics of strong mobility, high biological toxicity, and difficulty in removal after entering the food chain^3^. Cd^2+^ can cause a series of adverse effects on animals and plants, such as oxidative stress, photosynthesis inhibition, and liver damage^4^. Research has indicated that Cd^2+^ can lead to the abnormal electrocardiogram of zebrafish^5^. Additionally, Cd^2+^ can affect the activities of key enzymes, such as lipoprotein lipase and liver lipase, in liver metabolism, causing lipid and lipoprotein metabolism disorders and resulting in the fatty liver of fish^6^. Moreover, Cd^2+^ can inhibit plant growth and photosynthesis, interfering with ion metabolism and free radical formation^7^. Due to its strong ecological and toxicological effects, the control and remediation of Cd^2+^ pollution in water has attracted extensive attention. The removal of Cd^2+^ in water mainly includes physicochemical and bioremediation methods. Among these methods, adsorption has received extensive attention from researchers because of its simple operation, easy recovery and low cost^8,9^. Therefore, the selection of an adsorbent is essentially important for the removal of Cd^2+^ from water.

Biochar (BC) is a carbon-rich material obtained by the pyrolysis of biomass raw materials under anoxic and oxygen-limited conditions^10^. BC is often used as an adsorbent due to its large specific surface area and abundant functional groups. Previous studies have shown that BC prepared from different biomasses has a better removal effect on Cd^2+^ in water^11–13^. Northern China is a major corn-growing region. The comprehensive utilization of a large amount of corn stover has always been an urgent problem to be solved. Therefore, solving this problem by preparing corn stover into biochar and using it to remove Cd^2+^ from water is a very good technique. To prove the feasibility of this view in this research, Cd^2+^ is taken as the target pollutant and corn stover as the biomass material for biochar. Orthogonal experimental design is used to optimize the factors affecting the preparation of biochar (pyrolysis temperature, retention time, and heating rate) with the removal rate of Cd^2+^ in water as the target. In this test stage, the Cd^2+^ removal performance is compared to the original biochar and the acid/base-modified biochar under the corresponding preparation conditions. Only BC with a good Cd^2+^ removal effect is further studied. This decision not only greatly reduces the workload of the experiment but also makes the characterization of the samples more targeted; thus, the produced biochar has a better Cd^2+^ removal effect. Subsequently, adsorption isotherms and kinetics are studied, combined with a series of characterization methods, to more comprehensively reveal the mechanism of Cd^2+^ removal by corn stover biochar. This study provides a systematic theoretical basis and support for the promotion and application of biochar in the treatment of cadmium-containing wastewater.

## Materials and methods

### Chemicals and materials

All the chemicals in this experiment were obtained from Qinhuangdao Chemical Reagents Company (China) and were of analytical grade. We collected corn stover from local farmers, in Qinhuangdao, China.

### Preparation of corn stover biochar

After cleaning, the corn stover was dried at 70 °C for 10 h and then pulverized. Then, corn stover was pyrolysed into biochar in a tube furnace under a nitrogen atmosphere. The optimal preparation conditions were screened by a three-factor, four-level orthogonal experiment. Sixteen biochars prepared under different conditions were labelled C1, C2, C3, C4, C5, C6, C7, C8, C9, C10, C11, C12, C13, C14, C15, and C16. The orthogonal experiment is shown in Table 1. Finally, the pyrolytic biochar was ground with a mortar and passed through a 200-mesh standard sieve.

**Table 1:** Orthogonal experimental parameters.

Sample	Temperature (°C)	Retention time (h)	Heating rate (°C/min)
C1	400	1	5
C2	400	1.5	10
C3	400	2	15
C4	400	2.5	20
C5	500	1	10
C6	500	1.5	5
C7	500	2	20
C8	500	2.5	15
C9	600	1	15
C10	600	1.5	20
C11	600	2	5
C12	600	2.5	10
C13	700	1	20
C14	700	1.5	15
C15	700	2	10
C16	700	2.5	5

**Figure 1:**
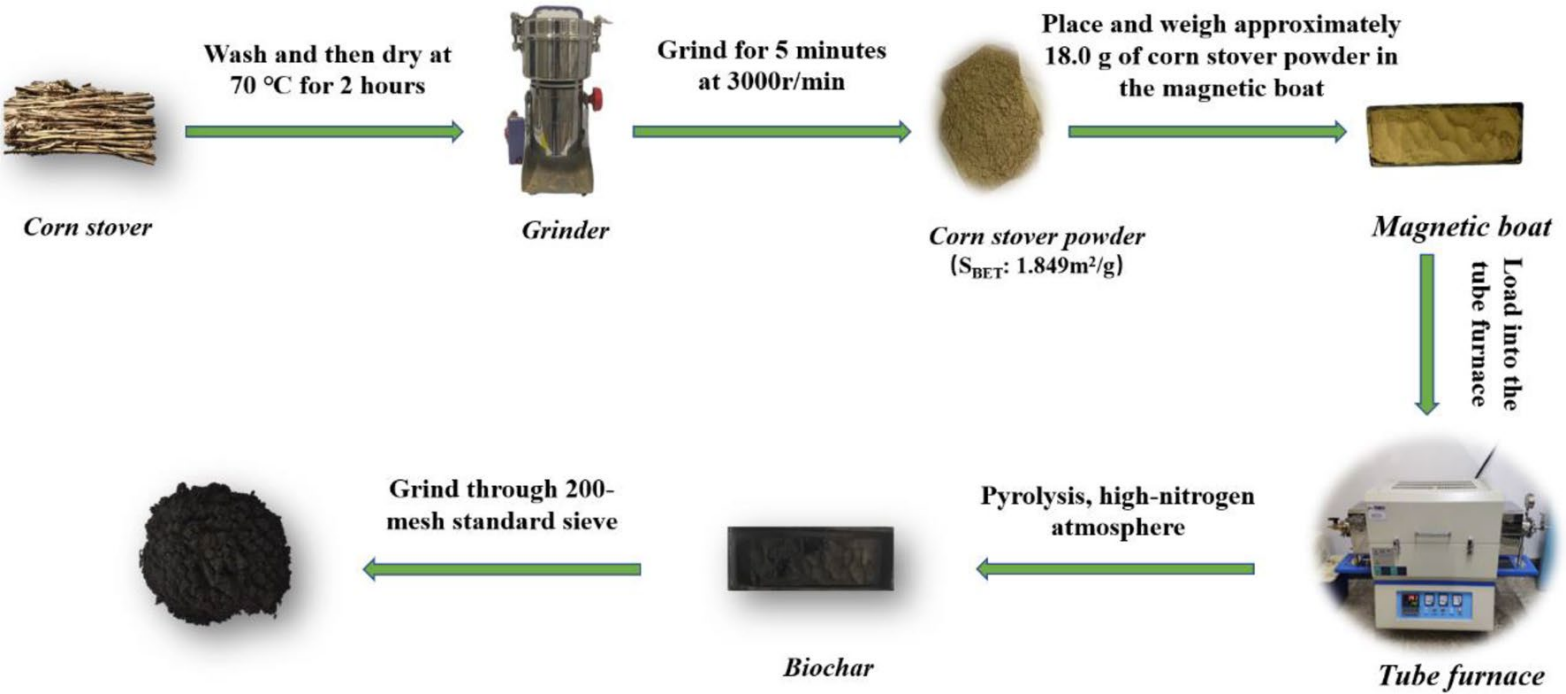
Preparation of corn stover biochar.

Biochar modification was as follows: the biochars were mixed with 1 mol/L HCl (or NaOH) at a ratio of 1:10 (w/v), followed by ultrasonication for 10 min, then pressure filtration, and finally, the sample was washed to a neutral pH. Corn stover biochars modified by acid/alkali were labelled BC-H/BC-OH, respectively. The biochar preparation process is shown in Fig. 1.

### Characterization of corn stover biochar

The S_BET_ of the biochar was measured by a specific surface area analyser (SSA-4000, BUILDER, China), and the functional groups of the biochar were determined by Fourier transform infrared spectroscopy (FTIR) (8400S, SHIMAZU, Japan). The apparent structure of the biochar was characterized by scanning electron microscopy (SEM) (Supra55 Sapphire, ZEISS, Germany and Coxem, OPTON, China). Thermogravimetric (TG) analysis of corn stover was performed using a thermogravimetric analyser (Setsy Evolution, SETARAM, France), and the surface phases of biochar were analysed by X-ray diffraction (XRD) (SmartLab (9), RIGAKU, Japan). Biochar yields were calculated as the ratio between the weights of the corn stover before and after pyrolysis. The sample was added to deionized water at a ratio of 1:20 (w/v), and the suspension was shaken for 1 h and allowed to stand for 5 min. The pH was measured by a pH meter. The surface functional groups of biochar were determined by improved Bohem titration^14,15^, and the cation exchange capacity (CEC) of biochar was measured by sodium acetate-flame photometry^14^.

### Adsorption experiment

#### Optimization of biochar

Cadmium chloride hydrate (CdCl_2_·2.5H_2_O) was dissolved to prepare the Cd^2+^ stock solution and then diluted to various concentrations for adsorption experiments. Corn stover biochars (0.20 g original, acid modified and base modified) were added into a 50 mL polyethylene centrifuge tube containing 20 mL Cd^2+^ solution (100 mg/L) and 0.01 mol/L CaCl_2_ as the background electrolyte. After shaking at 25 °C and 150 rpm for 24 h, the mixture was passed through a 0.45 μm filter membrane. Finally, the concentration of Cd^2+^ in the solution was determined by flame atomic absorption spectrometry (AA-6800F/G, Shimazu, Japan). The equilibrium adsorption capacity (q_e_, mg/g) of biochar was calculated based on the differences in the Cd^2+^ contents in the solution before and after adsorption. The removal rate (Rr, %) was calculated by the ratio of adsorption capacity to Cd^2+^ content before adsorption. The adsorption capacity and the removal rate of Cd^2+^ were calculated according to Eqs. (1) and (2), respectively. Through the experiment, the biochar with the best adsorption performance for Cd^2+^ was screened.

1 $$ q_{e} = \frac{{(C_{0} - C_{e} ) \times V}}{m} $$

2 $$ {\text{Rr}}({\text{\% }}) = \frac{{(C_{0} - C_{e} )}}{{C_{0} }} \times 100\% $$

#### Adsorption isotherm experiment

The biochar optimized in “Optimization of biochar” section was used for adsorption isotherm and adsorption kinetics experiments. Adsorption isotherm experiments were conducted with CdCl_2_ solutions of different initial concentrations (10, 50, 100, 200, 300, 400 and 500 mg/L). Other experimental steps were the same as those in “Optimization of biochar” section. The adsorption isotherm was drawn and fitted by Langmuir and Freundlich adsorption isotherm models^16^.

The Langmuir isotherm is as follows (Eq. 3):

3 $$ q_{e} = \frac{{q_{max} K_{L} C_{e} }}{{1*K_{L} C_{e} }} $$

where *q*_max_ (mg/g) is the maximum adsorption capacity of the adsorbent at equilibrium, *C*_e_ (mg/L) is the equilibrium concentration of adsorbate, and *K*_L_ (L/mg) is the Langmuir isotherm constant.

The Freundlich isotherm is as follows (Eq. 4):

4 $$ q_{e} = K _{F} C_{e}^{1/n} $$

where *K*_F_ (mg^1−n^ g^−1^ L^−n^) is the Freundlich isotherm constant and *n* is a constant.

#### Adsorption kinetics experiment

The concentration of Cd^2+^ was measured at 13 points in time from mins to 1440 min. The adsorption experiment method was the same as that in “Optimization of biochar”. The adsorption capacity of biochar on Cd^2+^ in water at different time points was fitted by pseudo-first-order (Eq. 5), pseudo-second-order (Eq. 6), Elovich (Eq. 7), and Webber–Morris intraparticle diffusion models (Eq. 8)^16–18^:

5 $$ q_{t} = q_{e} (1 - e^{{ - k_{1} t}} ) $$

6 $$ q_{t} = \frac{{k_{2} q_{e}^{2} t}}{{1 + q_{e} t}} $$

7 $$ q_{t} = 1{/}b\ln (ab) + 1{/}b\,ln\,t $$

8 $$ q_{t} = K_{id} t^{0.5} + C_{i} $$

where *q*_t_ (mg/g) is the amount of adsorbate at time *t*, *K*_1_ (h^−1^) and *K*_2_ (g mg^−1^ h^−1^) are the pseudo-first-order and pseudo-second-order reaction rate constants, respectively, *a* (mg g^−1^ min^−1^) and *b* (mg/g) are the initial adsorption rate and the desorption constant of the Elovich model, respectively, *t* (h) is the reaction time, *K*_id_ (mg g^−1^ min^−0.5^) is the intraparticle diffusion rate constant, and *C*_i_ is a constant.

### Factors influencing Cd^2+^ adsorption by biochar

The biochar optimized in “Optimization of biochar” section was used to explore the optimal adsorption conditions. In this study, the experimental steps were the same as those in “Optimization of biochar” section, only increasing the Cd^2+^ solution to 25 mL.

#### Electrolyte solution concentration

The concentrations of the electrolyte CaCl_2_ solution were 0.01, 0.04, 0.08, 0.12, 0.16, and 0.3 mol/L, and the other factors remained unchanged. The optimal concentration of electrolyte solution was determined according to the removal rate of Cd^2+^.

#### Initial pH value

Based on the research in “Electrolyte solution concentration” section, the influence of the initial pH value on the Cd^2+^ removal rate was further discussed. We used 0.1 mol/l HCl or NaOH to adjust the initial pH values to 2, 3, 4, 5, 6, and 7.

#### Biochar dosage

Combined with the optimal electrolyte concentration and pH, the influence of biochar addition on the Cd^2+^ removal rate was revealed. The biochar additions were 0.1, 0.2, 0.3, 0.4, 0.5 and 0.6 g.

## Results and discussion

### Thermogravimetric/differential thermogravimetry analyses of corn stover

**Figure 2:**
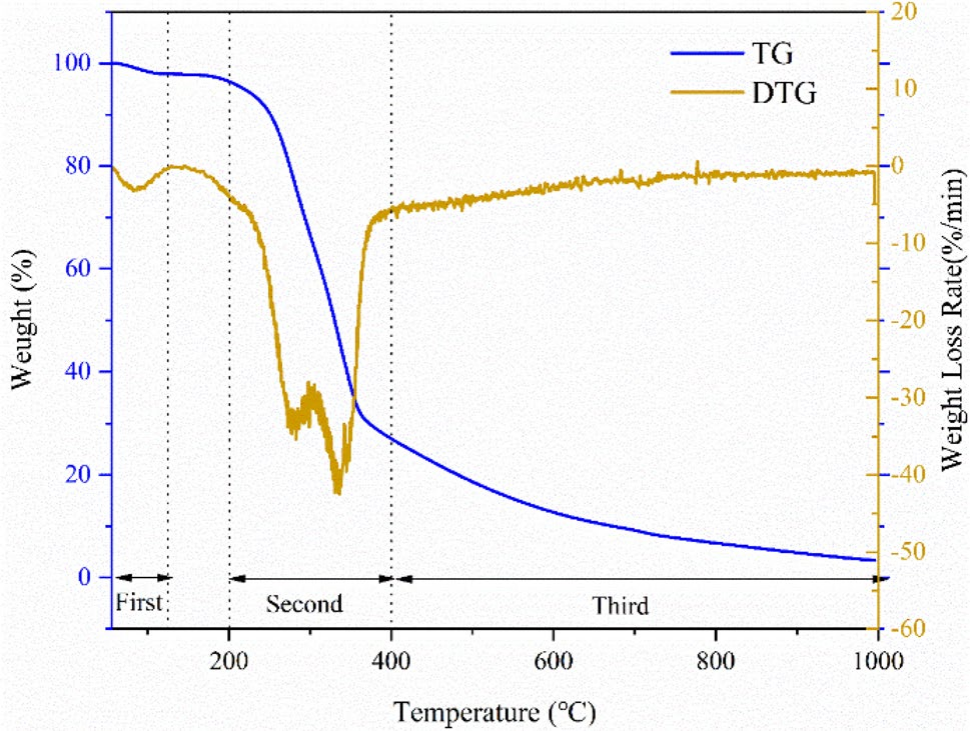
TG/DTG curves of corn stover.

Thermogravimetric/Differential Thermogravimetry (TG/DTG) curves are shown in Fig. 2. The pyrolysis process of corn stover could be divided into three stages. The first stage was the dehydration stage, which occurred at approximately 55–125 °C, and the weight loss was mainly accounted for by water^19^. The second stage was the pyrolysis stage, which occurred at approximately 200–400 °C and mainly involved the decomposition of cellulose, hemicellulose and a small amount of lignin. This process involved the generation of CO and CO_2_ and the breaking of carbonaceous polymer bonds^20^. In addition, a shoulder peak in the range of 265 to 300 °C in the DTG diagram could be caused by side chain decomposition and glycosidic bond cleavage of xylan during the pyrolysis of corn stover^21^. The third stage was the carbonization stage, which occurred above 400 °C; this stage mainly involved the decomposition of lignin^22,23^. The carbonization process was relatively slow after 600 °C; this process was called the passive pyrolysis stage^24^. In general, the TG loss in the pyrolysis process of corn stover was mainly from the moisture in the biomass sample in the first stage. Hemicellulose and cellulose decomposition occurred in the second stage, and lignin decomposition occurred in the third stage^25^. In this experiment, the minimum pyrolysis temperature for the preparation of biochar was 400 °C. Therefore, the pyrolysis of biochar was relatively complete.

### Characterization of biochar

#### Yield and specific surface area analyses

**Figure 3:**
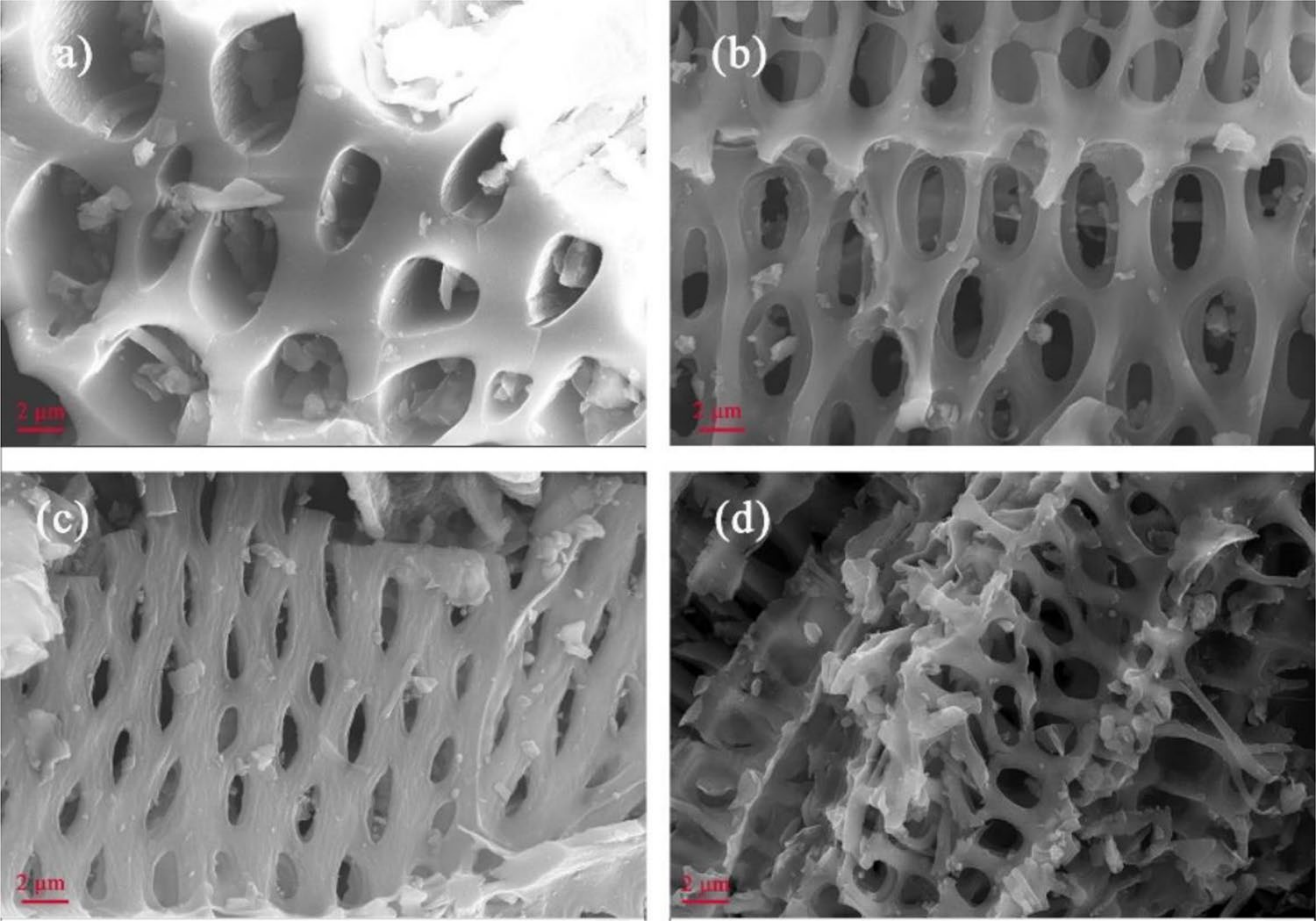
SEM (ZEISS) images of biochar at different pyrolysis temperatures: (**a**) C1, (**b**) C8, (**c**) C12, and (**d**) C16.

**Figure 4:**
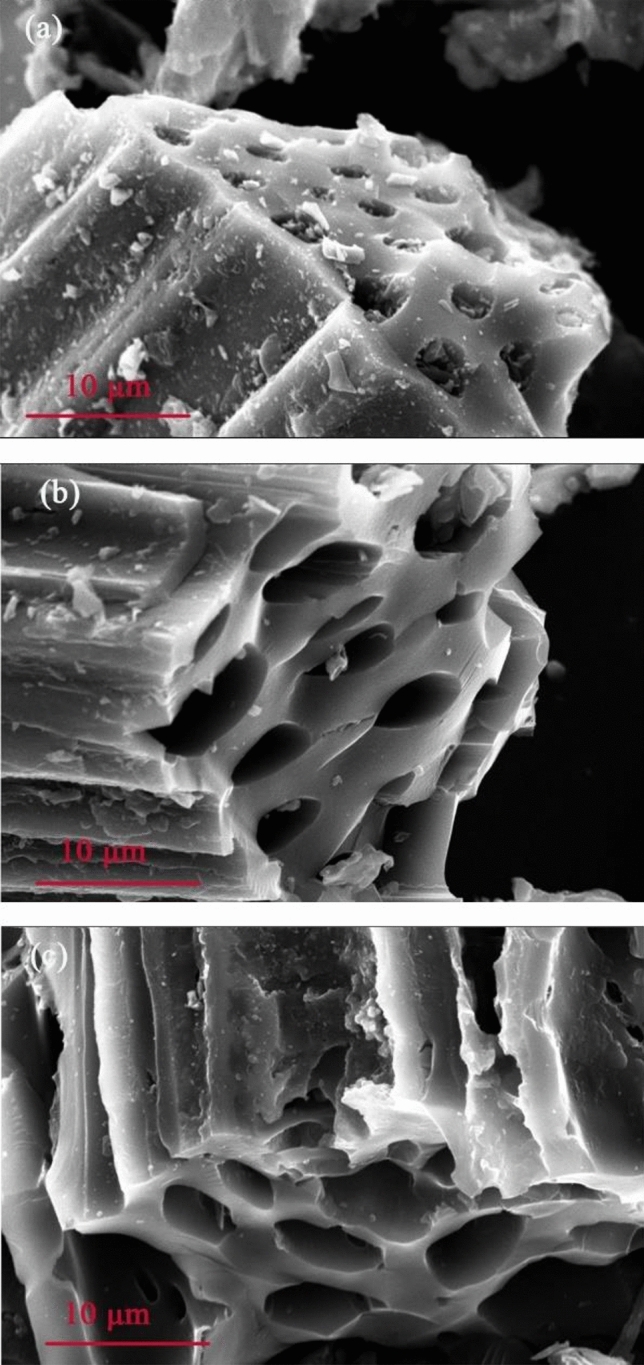
SEM (OPTON) images of C16 biochar and its acid/base modification: (**a**) C16, (**b**) C16-H, and (**c**) C–OH.

The yield and S_BET_ are presented in Table 2. BC, BC-H and BC-OH represent the origin, acid-modified, and base-modified biochar, respectively. The yield of corn stover biochar exhibited a negative correlation with the temperature and decreased from 39.65 to 28.26% when the pyrolysis temperature increased from 400 to 700 °C. This phenomenon could have occurred due to the loss of more volatile substances and the thermal degradation of lignocellulose with increasing temperature, thus reducing the yield of biochar^26,27^. The S_BET_ of the original biochar showed little difference below 700 °C but increased significantly at 700 °C. Combined with the SEM analysis (Fig. 3), at low temperatures, more ashes on the surface of biochar could block its pores so that the change in S_BET_ was not obvious. At 700 °C, because the ash content significantly reduced and the pyrolysis was more sufficient, the pores of the biochar were more developed, and the S_EBT_ significantly increased. The S_BET_ of the acid/base-modified biochar increased with increasing temperature. The S_BET_ of biochar was larger than that of the original biochar after acid and base modification at 400–600 °C. This phenomenon occurred because the porous structure of biochar was enhanced by acid and base modification^28^. Moreover, pickling removed most of the inorganic substances in biochar and reduced ash content, while alkali washing removed the tar on the surface of biochar to a certain extent^29^. However, at 700 °C, the S_BET_ of biochar after acid/base modification was lower than that of the original biochar. Combined with the SEM (Fig. 4), the acid/base modification caused the nanopores of biochar to collapse into mesopores or macropores^30^. Therefore, the well-developed pore structure of the biochar prepared at 700 °C was destroyed by acid/base modification, resulting in a significant decrease in S_BET_.

**Table 2:** Yield and S_BET_ of different biochars.

Sample	Yield (%)			S_BET_ (m^2^/g)		
	BC	BC-H	BC-OH	BC	BC-H	BC-OH
C1	38.104	32.477	36.322	3.960	6.050	5.512
C2	39.653	31.953	37.917	4.143	6.601	5.734
C3	35.221	31.791	32.563	4.334	7.277	6.284
C4	35.472	30.438	32.431	4.401	5.998	5.766
C5	32.106	28.583	30.639	4.554	11.899	6.179
C6	33.701	31.023	32.042	3.849	12.597	5.231
C7	30.268	26.657	29.313	4.679	22.027	6.457
C8	31.864	28.054	29.083	4.780	15.927	5.618
C9	29.319	26.442	26.628	3.745	39.018	6.53
C10	29.103	26.133	29.141	4.045	20.546	7.621
C11	30.622	27.989	28.427	4.228	20.496	10.644
C12	30.047	27.053	28.372	3.792	33.216	8.494
C13	28.258	24.855	24.913	6.271	14.047	6.333
C14	28.653	26.203	27.224	30.439	25.261	8.948
C15	28.391	25.534	26.766	81.682	17.684	58.25
C16	30.024	27.171	28.135	52.156	17.469	25.305

#### Scanning electron microscopy analysis

The C1, C8, C12 and C16 biochars had the highest Cd^2+^ removal rates at 400, 500, 600 and 700 °C, respectively. Therefore, these BCs were selected for SEM analysis. Figure 3 clearly showed that as the pyrolysis temperature increased from 400 to 700 °C, the pore structure of biochar became more developed, with a smaller pore size and more pores. Although there were numerous pores at 500 °C, the pores were not fully developed and were blocked inside. At 700 °C, the skeleton structure appeared, and the particle size of ash blocked in the pores decreased.

By taking C16 biochar with the highest removal rate of Cd^2+^ as the research object, the changes in the biochar surface before and after modification were compared. C16-H and C16-OH represent acid-modified and base-modified biochar, respectively. After acid/base modification, the ash content on the surface of the biochar decreased, and the pore size increased (Fig. 4). Therefore, some skeleton structures could collapse after corrosion, which was consistent with the previous S_BET_ results. Sun et al. discovered that citric acid-modified biochar would lead to micropore wall collapse and micropore loss, resulting in a reduction in S_BET_^31^. This finding was in agreement with the results of our study.

#### Fourier transform infrared spectroscopy analysis

**Figure 5:**
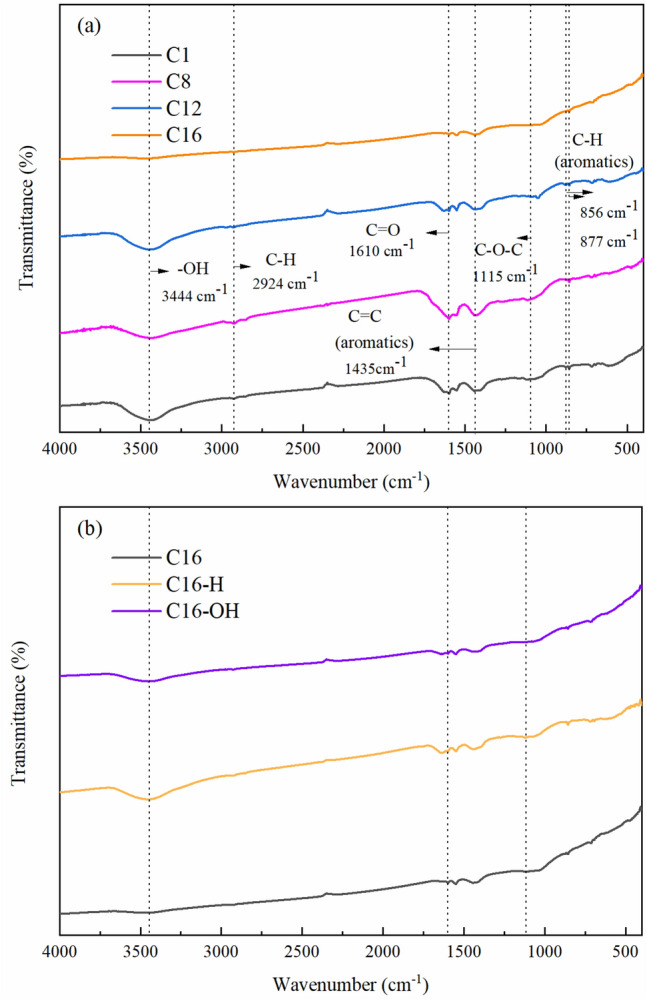
FTIR spectra of corn stover biochar: (**a**) different pyrolysis temperatures and (**b**) different modification treatments.

The FTIR spectra of biochar at different pyrolysis temperatures are presented in Fig. 5a.

As the pyrolysis temperature increased from 300 to 700 °C, the absorption peak intensity showed a downwards trend. There was a remarkable decrease in features associated with stretch O–H (3400 cm^−1^)^32^. The vibration peaks of C–H (2924 cm^−1^) and C=O (1610 cm^−1^) decreased with increasing temperature, which could be due to the reduction in –CH_2_ and –CH_3_ groups of small molecules and the pyrolysis of C=O into gas or liquid byproducts at high temperatures^33^. In addition, the peak at 1435 cm^−1^ was identified as the vibration of C=C bonds belonging to the aromatic skeleton of biochar. A decrease in the absorbance peaks was found at 1115 cm^−1^, which corresponded to C–O–C bonds. The ratio of intensities for C=C/C=O (1550–1650 cm^−1^) and C–O–C (1115 cm^−1^) to the shoulder (1100–1200 cm^−1^) gradually decreased, and the loss of –OH at 3444 cm^−1^ indicated that the oxygen content in biochar reduced. The cellulose and wood components were dehydrated, and the degree of biochar condensation increased at higher temperatures. The bending vibration peaks of Ar–H at 856 and 877 cm^−1^ changed little at different temperatures, which showed that the aromatic rings were relatively stable below 700 °C^34^. Combined with the above analysis the condensation degree of biochar increased gradually above 400 °C^35,36^. In summary, as the pyrolysis temperature increased, the degree of aromatization of biochar improved, and the numbers of oxygen-containing functional groups decreased continuously.

Figure 5b showed that after acid/base modification, the absorbance peaks at 3444 cm^−1^, 1610 cm^−1^ and 1115 cm^−1^ increased, indicating that the number of oxygen-containing functional groups increased. However, the stretching vibration peak of aromatic ring skeleton C=C (1435 cm^−1^) and the bending vibration peaks of Ar–H (856–877 cm^−1^) changed little. The number of functional groups of acid-modified biochar increased more than that of alkali-modified biochar. Mahdi et al. found that acid modification increased the number of functional groups in a study of biochar modification^37^. After acid/base modification, the number of oxygen-containing functional groups, such as hydroxyl and carboxyl groups, increased.

### Optimization of biochar

**Figure 6:**
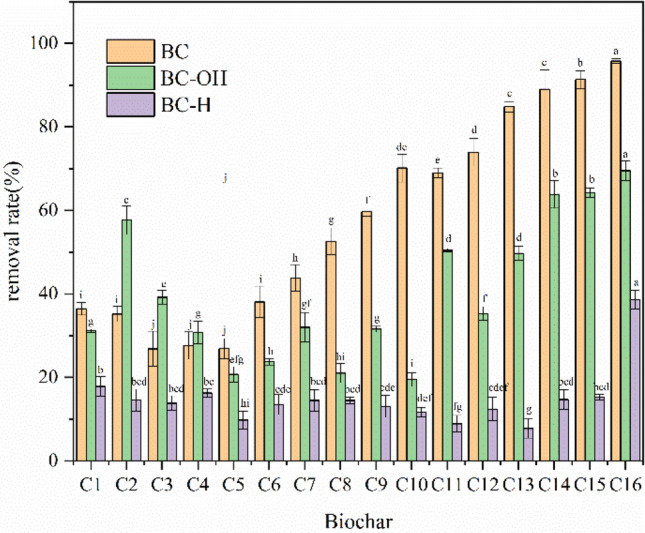
Cd^2+^ removal rate of different biochars (BC: original biochar, BC-OH: alkali-modified biochar, and BC-H: acid-modified biochar).

Figure 6 illustrates that the removal rates of Cd^2+^ by corn stover biochar (original, acid-modified, and base-modified biochars) consistently increased with increasing pyrolysis temperature. The highest removal rate reached 95.79% at 700 °C. The removal rate decreased after modification, especially after pickling. The results showed that C16 biochar had the best removal effect on Cd^2+^.

Intuitive and variance analyses were employed to explore the influences of biochar preparation conditions on the removal rate of Cd^2+^.

**Figure 7:**
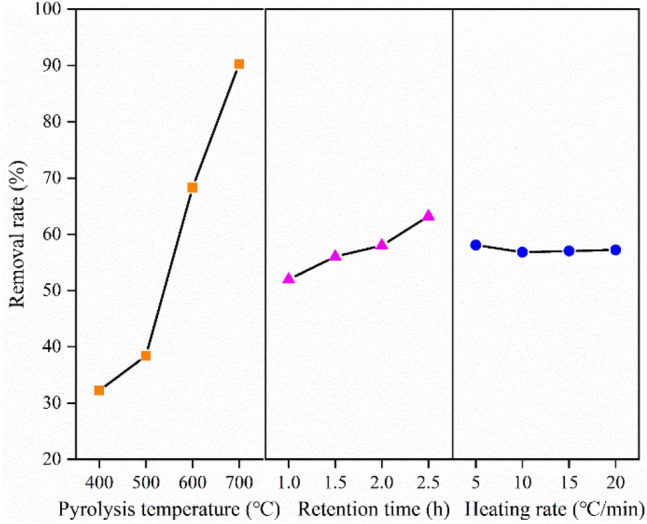
Intuitive analysis diagram of influencing factors for biochar preparation.

1. Intuitive analysis
  The intuitive analysis of the orthogonal experiment is shown in Table 3 and Fig. 7. The pyrolysis temperature had the most significant influence on the removal of Cd^2+^, followed by the retention time and finally the heating rate. Therefore, the optimal conditions for biochar preparation were a pyrolysis temperature of 700 °C, a retention time of 2.5 h, and a heating rate of 5 °C/min.
2. Variance analysis
  Variance analysis showed that the effect of pyrolysis temperature on the removal rate of Cd^2+^ was very significant (Table 4). The effects of retention time and heating rate were not significant. This phenomenon was consistent with the conclusions obtained in the intuitive analysis.

**Table 3:** Intuitive analyses of influencing factors of biochar preparation.

Sample	Pyrolysis temperature (°C) A	Retention time (h) B	Heating rate (°C/min) C	Removal rate (%)
C1	400	1	5	36.42
C2	400	1.5	10	35.17
C3	400	2	15	26.86
C4	400	2.5	20	30.47
C5	500	1	10	26.87
C6	500	1.5	5	30.24
C7	500	2	20	43.82
C8	500	2.5	15	52.62
C9	600	1	15	59.72
C10	600	1.5	20	69.76
C11	600	2	5	69.98
C12	600	2.5	10	73.92
C13	700	1	20	84.85
C14	700	1.5	15	89.00
C15	700	2	10	91.37
C16	700	2.5	5	95.79
Average value 1	32.230	51.965	58.108	
Average value 2	38.388	56.043	56.833	
Average value 3	68.345	58.008	57.050	
Average value 4	90.253	63.200	57.225	
Range	50.023	11.235	1.275	
Factor priority	A > B > C			
Best combination	A_4_B_4_C_1_

**Table 4:** Variance analysis.

Element	Sum of square of deviations	Degree of freedom	F ratio	F critical value	Significance
Pyrolysis temperature (°C)	8776.187	3	13.361	4.760	**
Retention time (h)	261.416	3	0.398	4.760	
Heating rate (°C/min)	3.755	3	0.006	4.760	
Error	1313.750	6			

*P* < 0.01 indicates a very significant difference represented by **.

### Analysis of adsorption mechanism

The S_BET_ of the unmodified biochar did not change significantly with temperature, which indicated that S_BET_ could potentially not be a critical factor for Cd^2+^ adsorption. Qi et al. obtained a similar conclusion when studying the adsorption of Cd^2+^ in water by chicken litter biochar^38^. In addition to S_BET_, the four primary mechanisms involved in the removal of heavy metal ions by biochar were as follows: (1) Ion exchange: the alkali or alkaline earth metals in biochar (K^+^, Ca^2 +^, Na^+^, and Mg^2+^) were the dominant cations in ion exchange^39^. (2) The complexation of oxygen-containing functional groups mainly included hydroxyl and carboxyl groups^40^. (3) Mineral precipitation: Cd^2+^ was precipitated by minerals on the surface of biochar to form Cd_3_(PO_4_)_2_ and CdCO_3_^41^. Soluble cadmium precipitated with some anions released by biochar, such as CO_3_^2−^, PO_4_^3−^ and OH^−^^42,43^. (4) π electron interaction: Cd^2+^ coordinated with the π electrons of C=C or C=O at low pyrolysis temperatures^43,44^. Biochar contains more aromatic structures at high pyrolysis temperatures, which could provide more π electrons. Therefore, the π electron interaction in adsorption of Cd^2+^ was effectively enhanced^45^.

C1, C8, C12 and C16 were selected to study the adsorption mechanism. Related physicochemical properties are given in Table 5.

**Table 5:** Physicochemical properties of biochar at different pyrolysis temperatures.

Sample	CEC (cmol/kg)	pH	Acid functional groups (mmol/g)	Alkaline functional groups (mmol/g)
C1	45.763 ± 0.133d	7.86 ± 0.03d	1.030 ± 0.027a	1.325 ± 0.032a
C8	47.518 ± 0.124c	9.61 ± 0.07c	0.651 ± 0.013b	1.625 ± 0.028b
C12	52.212 ± 0.341b	10.24 ± 0.04b	0.621 ± 0.021bc	1.652 ± 0.023b
C16	44.164 ± 0.262a	11.09 ± 0.03a	0.613 ± 0.010c	1.791 ± 0.048a

Different letters (a, b, c, d, e) represent significant differences (*p* < 0.05) between treatments in same physicochemical properties.

The CEC of biochar gradually increased as the pyrolysis temperature increased, reaching a maximum at 600 °C and slightly decreasing at 700 °C. This phenomenon could have occurred because the crystalline minerals under high pyrolysis temperatures inhibited the exchange of cations on the surface of biochar with Cd^2+^ in aqueous solution^46^. Nevertheless, CEC did not change significantly with temperature; thus, CEC was not the main adsorption mechanism. With increasing pyrolysis temperature, the number of acidic functional groups decreased gradually, while the number of alkaline functional groups increased. The main functional groups used to remove Cd^2+^ were generally considered acidic oxygen-containing functional groups. However, the number of these functional groups decreased with increasing pyrolysis temperature, which weakened the complexation on the surface of the biochar. However, this result was contradictory to the results of Cd^2+^ adsorption. Therefore, the functional groups were not the main adsorption mechanism.

To further explore the adsorption mechanism of Cd^2+^, the biochar before and after the adsorption of Cd^2+^ was characterized by XRD. As shown in Fig. 7a, C16-100Cd and C16-200Cd represented the biochar after Cd^2+^ adsorption when the concentrations of cadmium solution were 100 mg/l and 200 mg/l, respectively. The results showed that new peaks appeared at 30.275° and 36.546° after adsorption, corresponding to CdCO_3_. The spike at 29.454° was due to Cd(OH)_2_. Additionally, the intensity of the CdCO_3_ peak increased significantly from C16-100Cd to C16-200Cd, indicating that mineral precipitation occurred in adsorption. Liu et al. found similar results in a study on removing Cd^2+^ from water by blue algae biochar^12^. However, as the concentration of Cd^2+^ increased from 0 to 200 mg/L, the diffraction peak at 2θ = 29.454° first increased and then decreased. This because the peak position of CaCO_3_ at 2θ = 29.369° was very close to Cd(OH)_2_ at 2θ = 29.454°. At low concentrations, the production of Cd(OH)_2_ was greater than that of CdCO_3_. When the initial concentration of Cd^2+^ increased, more CO_3_^2−^ released by CaCO_3_ combined with Cd^2+^ to form CdCO_3_, resulting in a reduction in the diffraction peak.

**Figure 8:**
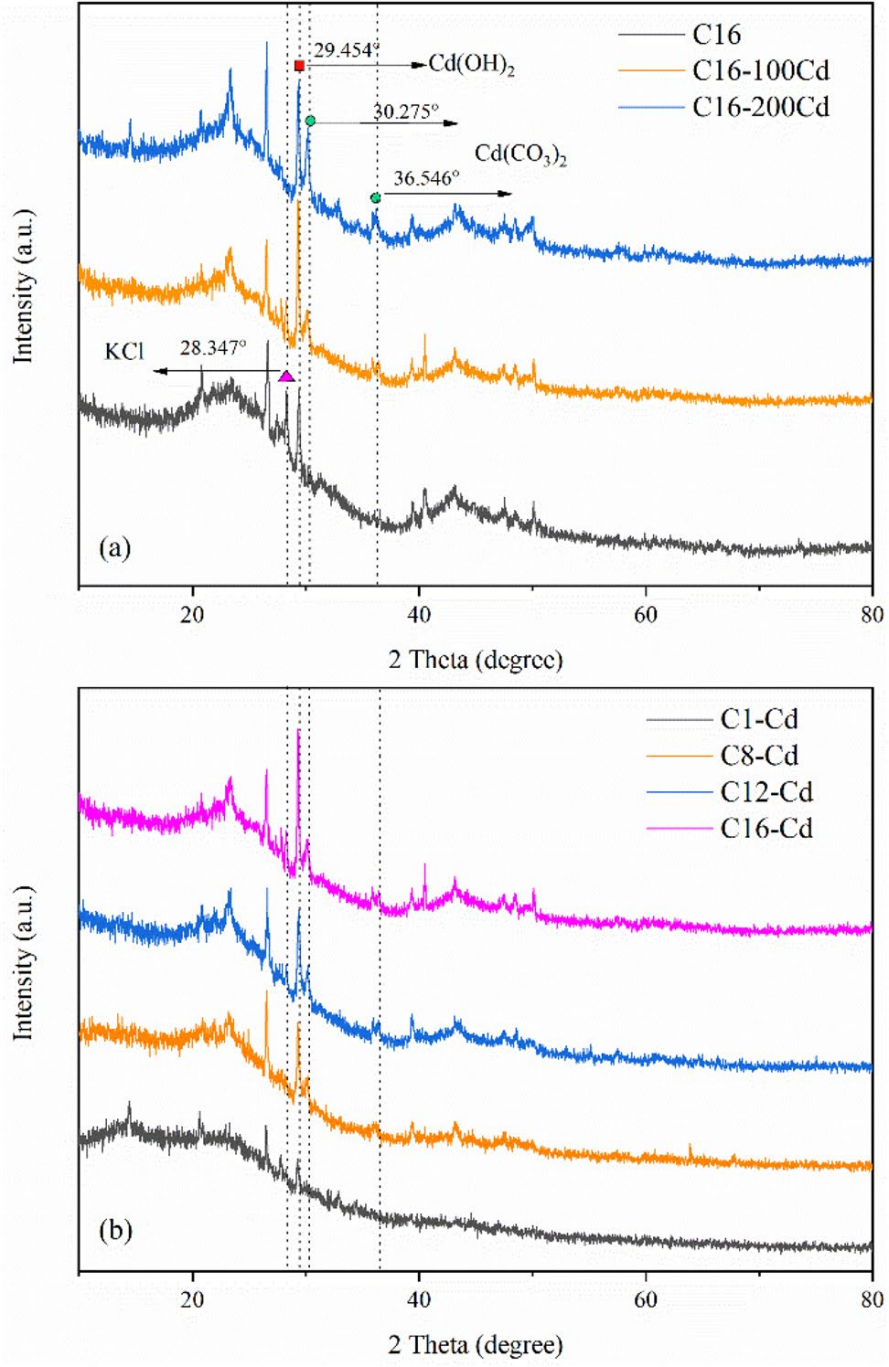
XRD images: (**a**) before and after adsorption of Cd^2+^ on C16 biochar and (**b**) Cd^2+^ adsorption by biochar at different pyrolysis temperatures.

As presented in Fig. 8b, the peak intensities of CdCO_3_ and Cd(OH)_2_ gradually increase with increasing pyrolysis temperature. On the one hand, this phenomenon could be ascribed to the increase in the mineral content of biochar with increasing pyrolysis temperature. On the other hand, the pH value of biochar increased with increasing pyrolysis temperature. In this way, more OH^−^ was released, thus forming more Cd(OH)_2_. Wang et al. obtained similar results^42^. Moreover, the peak intensity of KCl at 2θ = 28.347° decreased after adsorption, as shown in Fig. 8a, which indicated that ion exchange took part in adsorption.

In addition, the FTIR spectra showed that the number of functional groups, such as C=C and C=O, in biochar decreased with increasing pyrolysis temperature, leading to the weakening of cation–π interactions between Cd^2+^ and C=C and C=O. In contrast, due to the enhanced aromatization of functional groups on the surface of biochar, many lone pair electrons existed in the electron-rich domains of the graphene-like structure, which in turn enhanced the cation–π interactions. Harvey et al., based on the study of Cd^2+^ adsorption by plant biochar, concluded that the electron-rich domain bonding mechanism between Cd^2+^ and the graphene-like structure on the surface of biochar played a more significant role in biochar with a high degree of carbonization^45^. Therefore, π-electron interactions could play a dominant role in Cd^2+^ adsorption on high-temperature pyrolysis biochar. Moreover, the results showed that the number of alkaline functional groups increased while acidic functional groups decreased with the increase in pyrolysis temperature. It is generally believed that acidic functional groups could withdraw electrons, and basic functional groups could donate electrons^47,48^. The biochar with higher pyrolysis temperature contained more alkaline functional groups, which improved the electron donating ability of biochar and enhanced the cation–π electron effect.

In summary, mineral precipitation and π electron coordination were the main mechanisms of removing Cd^2+^ from water by corn stover biochar. This phenomenon explained why the Cd^2+^ removal rate of acid/base–modified biochar decreased. After modification, the functional groups on the surface of biochar increased, but the inorganic minerals were removed. Pickling resulted in the loss of soluble minerals and alkaline functional groups on the surface of biochar, which was not conducive to adsorption^49^. After alkaline washing, more PO_4_^3−^, CO_3_^2−^ and HCO_3_^−^ were released, thereby reducing the mineral precipitation^50,51^. Since NaOH had a weaker destructive effect than HCl and introduced some OH^−^, alkaline washing had little effect on the removal rate of Cd^2+^.

### Adsorption isotherm and adsorption kinetics

#### Adsorption isotherm

**Figure 9:**
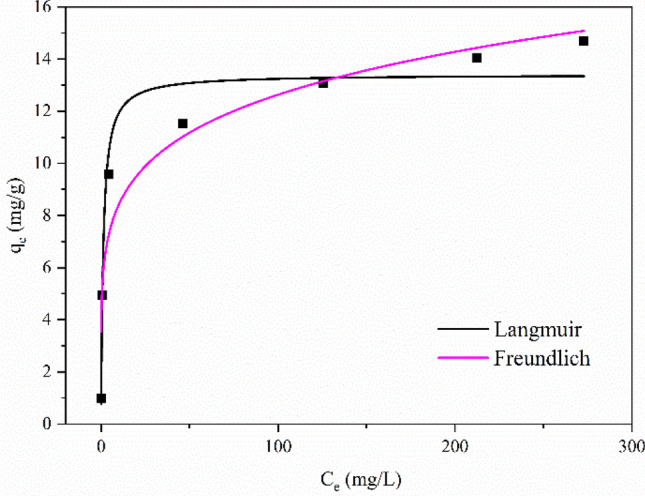
Adsorption isotherm.

The adsorption isotherms were fitted with Langmuir (Eq. 3) and Freundlich (Eq. 4) models, as shown in Fig. 9, and the fitting parameters are listed in Table 6.

**Table 6:** Fitting parameters of the adsorption isotherm model.

Langmuir			Freundlich		
*q*_max_ (mg/g)	*K*_L_ (L/mg)	*R*^2^	*K*_F_ (mg^1−n^ g^−1^ L^−n^)	*n*^−1^	*R*^2^
13.404	0.803	0.963	5.600	0.177	0.919

The Langmuir model (*R*^2^ > 0.963) was more suitable than the Freundlich model (*R*^2^ > 0.919), indicating that the adsorption sites of biochar were evenly distributed, and adsorption was mainly monolayer. Parameter *K*_L_ reflected the difficulty of adsorption and was generally divided into four types: unfavourable (*K*_L_ > 1), favourable (0 < *K*_L_ < 1), linear (*K*_L_ = 1), or irreversible (*K*_L_ = 0)^52,53^. The *K*_L_ values obtained by fitting were all between 0 and 1, suggesting that it was easy to adsorb. According to the fitting parameters of the Langmuir model, it could be inferred that the maximum adsorption capacity of corn stover biochar for Cd^2+^ was 13.4 mg/g. This result was higher than the maximum adsorption capacity of Cd^2+^ by biochar derived from oil seed rape, miscanthus and wheat in other studies (6.77, 11.33 and 12.35 mg/g, respectively)^54^. The maximum adsorption capacity of Hickory wood biochar before and after sodium hydroxide modification for Cd^2+^ was 0.2 mg/g and 0.98 mg/g, respectively^55^, which was lower than the biochar derived from corn stover in this study.

#### Adsorption kinetics

**Figure 10:**
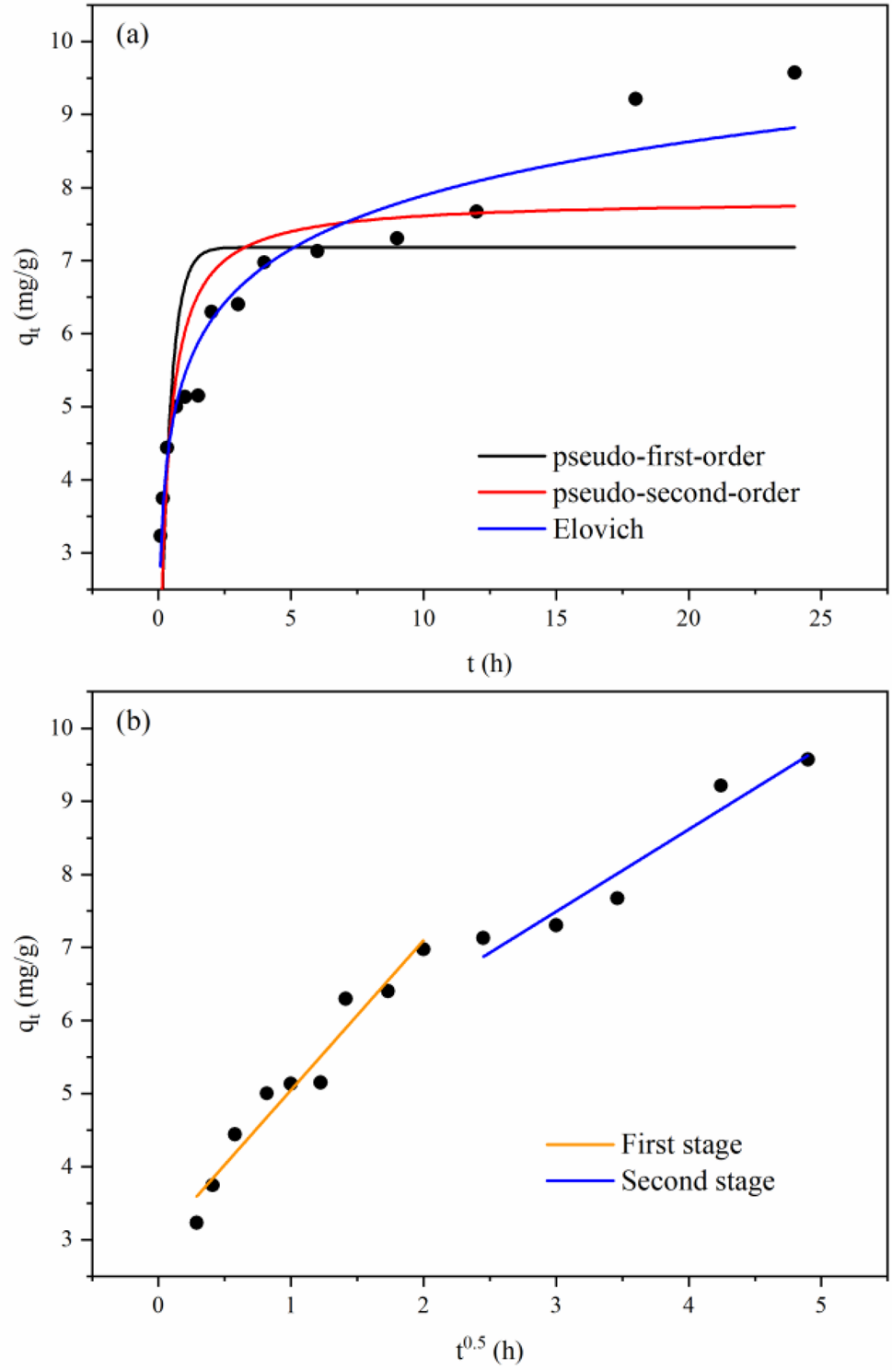
Fitting curves of the pseudo-first-order, pseudo-second-order and Elovich models (**a**) and intraparticle diffusion model (**b**).

Pseudo-first-order (Eq. 5), pseudo-second-order (Eq. 6) and Elovich (Eq. 7) models were employed to fit the adsorption kinetics process, and the results are presented in Fig. 10a and Table 7.

**Table 7:** Fitting parameters of the pseudo-first-order, pseudo-second-order and Elovich models.

Pseudo-first-order			Pseudo-second-order			Elovich		
*q*_e_ (mg/g)	*K*_1_ (h^−1^)	*R*^2^	*q*_e_ (mg/g)	*K*_2_ (g mg^−1^ h^−1^)	*R*^2^	*(b*^−1^*)ln(ab)*	*b*^−1^	*R*^2^
7.184	2.636	0.509	7.843	0.425	0.725	5.450	1.061	0.944

According to the fitting parameters of the kinetic model, the fitting effect of the pseudo-second-order model was better than that of the pseudo-first-order model. This phenomenon indicated that adsorption could be controlled by chemisorption, which could be roughly divided into two stages: rapid adsorption within 4 h and slow adsorption after 4 h. The adsorption capacity reached 6.98 mg/g at 4 h, accounting for 72.9% of the total adsorption capacity. In the fast adsorption stage, due to the existence of numerous active adsorption sites on the sample, the adsorption capacity increased significantly with time. With the decrease in the number of adsorption sites, the samples entered the slow stage, and the adsorption rate slowed and gradually approached equilibrium^56^. In comparison, the Elovich model had the best fitting effect on adsorption (*R*^2^ > 0.944), indicating that the adsorption of Cd^2+^ by corn stover biochar occurred by heterogeneous chemisorption^57^. The results were consistent with the previous adsorption mechanism.

The Webber–Morris intraparticle diffusion model (Eq. 8) is often used to predict the possibility of intraparticle diffusion^58^. The adsorption process could be divided into different stages according to the adsorption characteristics^59^. The Webber–Morris intraparticle diffusion model showed that adsorption consisted of two stages, as reflected in Fig. 10b. The first stage was the diffusion of Cd^2+^ to the surface of the biochar. The second stage was the adsorption of Cd^2+^ on biochar. Since *K*_1d_ was greater than *K*_2d_ (Table 8), the second stage was the control step of adsorption. Neither of the two fitting lines passed through the origin, indicating that intraparticle diffusion was not the only rate-determining step in adsorption^60^. The adsorption process could be affected by liquid film diffusion and the physicochemical interaction between Cd^2+^ and biochar. Similar results were obtained by Pholosi et al. using magnetite-coated biomass to adsorb Cr(VI)^60^.

**Table 8:** Fitting parameters of the Webber–Morris intraparticle diffusion model.

First stage of intraparticle diffusion			Second stage of intraparticle diffusion		
*K*_1d_ (mg g^−1^ min^−0.5^)	*C*_1_	*R*^2^	*K*_2d_ (mg g^−1^ min^−0.5^)	*C*_2_	*R*^2^
2.049	2.999	0.948	0.988	4.677	0.917

### Optimal conditions for Cd^2+^ adsorption by biochar

#### Electrolyte concentration

**Figure 11:**
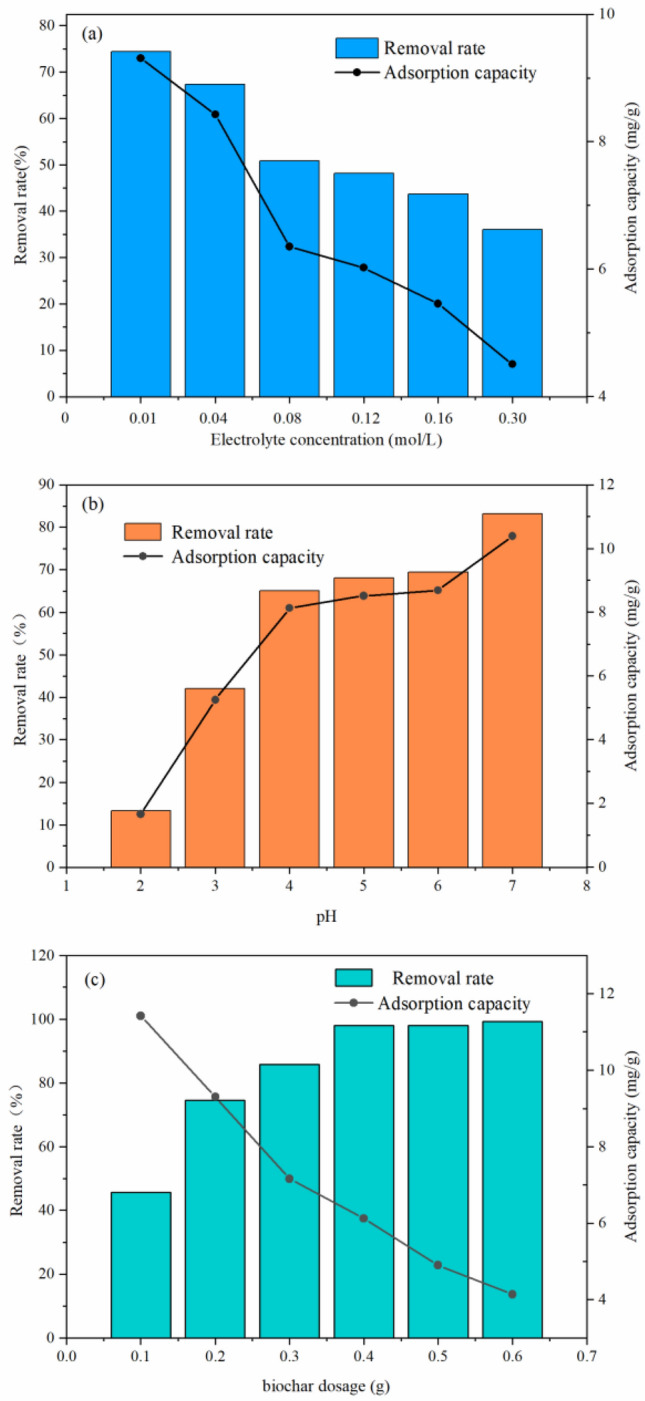
Effect of electrolyte concentration (**a**), pH value (**b**) and biochar dosage (**c**) on the Cd^2+^ removal rate.

Figure 11a showed that the electrolyte concentration was negatively correlated with the removal rate and adsorption capacity. The removal rate of Cd^2+^ decreased from 74.465 to 36.02% as the CaCl_2_ concentration increased from 0.01 to 0.3 mol/L, which could be caused by the competitive adsorption between Ca^2+^ and Cd^2+^ and the formation of a water-soluble metal–anion complex (CdCl^+^)^61,62^. Therefore, the removal rate of Cd^2+^ was the highest when the concentration of CaCl_2_ was 0.01 mol/L.

#### pH

As shown in Fig. 11b, the removal rate of Cd^2+^ improved significantly with the initial pH increase, and the upwards trend gradually slowed after the pH value reached 4, which could be explained by the competitive adsorption of Cd^2+^ and H^+^, the electrostatic repulsion between Cd^2+^ and the positive charge on the adsorbent surface. With the increase in pH and the decrease in protons, more binding sites were exposed, promoting the adsorption of Cd^2+^^12,63^. In addition, mineral precipitates dissolved at low pH values, affecting adsorption. The optimal pH value was 7. Considering the economic benefits, combined with the Cd^2+^ removal rate, the pH value should be selected in a neutral range.

#### Biochar dosage

The biochar dosage had an important effect on the adsorption of Cd^2+^ (Fig. 11c). As the biochar dosage increased, the removal rate of Cd^2+^ gradually increased to 97.96% and then stabilized. This phenomenon could have occurred because with the increase in biochar dosage from 0.1 to 0.4 g, the surface adsorption sites increased rapidly, thus promoting the adsorption of cadmium and increasing the removal rate of cadmium. When the biochar dosage was higher than 0.4 g, the removal rate did not increase, indicating that the Cd^2+^ in the solution reached adsorption equilibrium^64^. However, the adsorption capacity decreased from 11.42 to 4.14 mg/g with increasing biochar dosage. This phenomenon could have occurred because although the adsorption sites increased with the addition of biochar dosage, the amount of adsorbate remained constant; thus, the mass of Cd^2+^ adsorbed per unit mass of biochar decreased^65^. Additionally, due to the aggregation and overlap of adsorption sites, which could be caused by the increase in biochar dosage, the effective adsorption area decreased, and the diffusion path length increased, reducing the adsorption capacity^66^. Therefore, under the experimental concentration, the optimal addition amount of biochar was 0.4 g.

## Conclusion

This study demonstrated that corn stover biochar could effectively remove Cd^2+^ from water. The preparation conditions of corn stover biochar were optimized by the orthogonal test method. The results showed that when the pyrolysis temperature was 700 °C, the residence time was 2.5 h, and the heating rate was 5 °C/min, biochar with the highest removal rate of Cd^2+^ was produced. Under the same conditions, the acid/base-modified biochar did not improve the removal rate of cadmium compared to the original biochar. To further investigate the adsorption mechanism of cadmium in water by biochar, a series of chemical and characterization analyses (S_BET_, CEC, SEM, XRD, FTIR) were conducted, indicating that mineral precipitation and π electrons were the main removal mechanisms. Moreover, the isotherm and kinetic models were studied. The results showed that adsorption was consistent with the Langmuir adsorption isotherm, Elovich kinetic and Webber–Morris intraparticle diffusion models. The theoretical maximum adsorption capacity was 13.4 mg/g. In addition, to better understand the application conditions of biochar adsorption of Cd^2+^ in water, the optimal adsorption conditions were screened. When the concentration of CaCl_2_ was 0.01 mol/L, the pH was 7, and the amount of biochar was 0.4 g, the removal rate of Cd^2+^ by biochar was the highest. Although the natural water environment could be more complex and the application of corn stover biochar needs further investigation, this study provided a systematic and comprehensive theoretical basis for a better understanding of adsorption and the mechanisms of Cd^2+^ removal from water by corn stover biochar; this study provided a powerful data guarantee for the future application of corn straw biochar in the field of water pollution control.

## Data Availability

The raw data supporting the conclusions of this article are available from the corresponding author upon reasonable request.

## Abbreviations

SEM: Scanning electron microscopy
FTIR: Fourier transform infrared spectroscopy
XRD: X-ray diffractometer
TG/DTG: Thermogravimetric/Differential Thermogravimetry
S_BET_: Specific surface area
CEC: Cation exchange capacity
BC: Biochar
BC-H: Biochars modified by acid
BC-OH: Biochars modified by alkali

## References

[1] Martelli A, Rousselet E, Dycke C, Bouron A, Moulis J-M (2006). Cadmium toxicity in animal cells by interference with essential metals. Biochimie.

[2] Muntau H, Baudo R (1992). Sources of cadmium, its distribution and turnover in the freshwater environment. Iarc Sci. Publ..

[3] Zhou Q (2020). Cadmium adsorption to clay-microbe aggregates: implications for marine heavy metals cycling. Geochim. Cosmochim. Acta.

[4] Chen F, Gao J, Zhou Q (2012). Toxicity assessment of simulated urban runoff containing polycyclic musks and cadmium in Carassius auratus using oxidative stress biomarkers. Environ. Pollut..

[5] Xing N (2017). Cadmium stress assessment based on the electrocardiogram characteristics of zebra fish (Danio rerio): QRS complex could play an important role. Aquat. Toxicol..

[6] Liu X-J (2011). Antioxidant responses, hepatic intermediary metabolism, histology and ultrastructure in Synechogobius hasta exposed to waterborne cadmium. Ecotoxicol. Environ. Saf..

[7] Wahid A, Arshad M, Farooq M, Lichtfouse E (2010). Cadmium phytotoxicity: responses, mechanisms and mitigation strategies: a review. Organic Farming, Pest Control and Remediation of Soil Pollutants: Organic Farming, Pest Control and Remediation of Soil Pollutants.

[8] Lu M (2022). Potentiality of the porous geopolymer sphere in adsorption of Pb (II) from aqueous solutions: behaviors and mechanisms. Ceram. Int..

[9] McGinley J (2022). Batch adsorption of herbicides from aqueous solution onto diverse reusable materials and granulated activated carbon. J. Environ. Manag..

[10] Marzeddu S (2021). A life cycle assessment of an energy-biochar chain involving a gasification plant in Italy. Land.

[11] Xiang J, Lin Q, Yao X, Yin G (2021). Removal of Cd from aqueous solution by chitosan coated MgO-biochar and its in-situ remediation of Cd-contaminated soil. Environ. Res..

[12] Liu P, Rao D, Zou L, Teng Y, Yu H (2021). Capacity and potential mechanisms of Cd(II) adsorption from aqueous solution by blue algae-derived biochars. Sci. Total Environ..

[13] Boni M, Chiavola A, Antonucci A, Di Mattia E, Marzeddu S (2018). A novel treatment for Cd-contaminated solution through adsorption on beech charcoal: the effect of bioactivation. Desalin. Water Treat..

[14] Gaskin JW, Steiner C, Harris K, Das KC, Bibens B (2008). Effect of low-temperature pyrolysis conditions on biochar for agricultural use. Trans. Asabe.

[15] Goertzen SL, Thériault KD, Oickle AM, Tarasuk AC, Andreas HA (2010). Standardization of the Boehm titration. Part I. CO_2_ expulsion and endpoint determination. Carbon.

[16] Febrianto J (2009). Equilibrium and kinetic studies in adsorption of heavy metals using biosorbent: a summary of recent studies. J. Hazard. Mater..

[17] Jung K-W, Lee SY, Lee YJ (2018). Hydrothermal synthesis of hierarchically structured birnessite-type MnO_2_/biochar composites for the adsorptive removal of Cu(II) from aqueous media. Bioresour. Technol..

[18] Shenvi SS, Isloor AM, Ismail AF, Shilton SJ, Al Ahmed A (2015). Humic acid based biopolymeric membrane for effective removal of methylene blue and rhodamine B. Ind. Eng. Chem. Res..

[19] Mansaray KG, Ghaly AE (1999). Determination of kinetic parameters of rice husks in oxygen using thermogravimetric analysis. Biomass Bioenergy.

[20] Yang H, Yan R, Chen H, Lee DH, Zheng C (2007). Characteristics of hemicellulose, cellulose and lignin pyrolysis. Fuel.

[21] Shafizadeh F, McGinnis GD, Philpot CW (1972). Thermal degradation of xylan and related model compounds. Carbohydr. Res..

[22] Rony AH (2019). Kinetics, thermodynamics, and physical characterization of corn stover (Zea mays) for solar biomass pyrolysis potential analysis. Bioresour. Technol..

[23] Huang X, Yin H, Zhang H, Mei N, Mu L (2022). Pyrolysis characteristics, gas products, volatiles, and thermo-kinetics of industrial lignin via TG/DTG-FTIR/MS and in-situ Py-PI-TOF/MS. Energy.

[24] Ma F (2013). Thermogravimetric study and kinetic analysis of fungal pretreated corn stover using the distributed activation energy model. Bioresour. Technol..

[25] Mani T, Murugan P, Abedi J, Mahinpey N (2010). Pyrolysis of wheat straw in a thermogravimetric analyzer: effect of particle size and heating rate on devolatilization and estimation of global kinetics. Chem. Eng. Res. Des..

[26] Kumar Sakhiya A (2021). Copper(II) removal from aqua solution using rice straw derived biochar. Mater. Today Proc..

[27] Shakya A, Vithanage M, Agarwal T (2022). Influence of pyrolysis temperature on biochar properties and Cr(VI) adsorption from water with groundnut shell biochars: mechanistic approach. Environ. Res..

[28] Xue S (2021). Enhanced adsorption of Rhodamine B over Zoysia sinica Hance-based carbon activated by amminium chloride and sodium hydroxide treatments. Colloids Surf. A Physicochem. Eng. Asp..

[29] Regmi P (2012). Removal of copper and cadmium from aqueous solution using switchgrass biochar produced via hydrothermal carbonization process. J. Environ. Manag..

[30] Tseng R-L (2006). Mesopore control of high surface area NaOH-activated carbon. J. Colloid Interface Sci..

[31] Sun L, Chen D, Wan S, Yu Z (2015). Performance, kinetics, and equilibrium of methylene blue adsorption on biochar derived from eucalyptus saw dust modified with citric, tartaric, and acetic acids. Bioresour. Technol..

[32] Huang K (2022). Highly efficient removal of cadmium from aqueous solution by ammonium polyphosphate-modified biochar. Chemosphere.

[33] Zhuang Z, Wang L, Tang J (2021). Efficient removal of volatile organic compound by ball-milled biochars from different preparing conditions. J. Hazard. Mater..

[34] Wang L (2011). Preparation of carbon black from rice husk by hydrolysis, carbonization and pyrolysis. Bioresour. Technol..

[35] Lee JW (2010). Characterization of biochars produced from cornstovers for soil amendment. Environ. Sci. Technol..

[36] Zhu L (2015). Biochar of corn stover: Microwave-assisted pyrolysis condition induced changes in surface functional groups and characteristics. J. Anal. Appl. Pyrolysis.

[37] Mahdi Z, El Hanandeh A, Yu QJ (2019). Preparation, characterization and application of surface modified biochar from date seed for improved lead, copper, and nickel removal from aqueous solutions. J. Environ. Chem. Eng..

[38] Qi F (2017). Thermal stability of biochar and its effects on cadmium sorption capacity. Bioresour. Technol..

[39] Mohan D (2007). Sorption of arsenic, cadmium, and lead by chars produced from fast pyrolysis of wood and bark during bio-oil production. J. Colloid Interface Sci..

[40] Sun J, Lian F, Liu Z, Zhu L, Song Z (2014). Biochars derived from various crop straws: characterization and Cd(II) removal potential. Ecotoxicol. Environ. Saf..

[41] Zhang F (2015). Efficiency and mechanisms of Cd removal from aqueous solution by biochar derived from water hyacinth (Eichornia crassipes). J. Environ. Manag..

[42] Wang Z (2015). Investigating the mechanisms of biochar’s removal of lead from solution. Bioresour. Technol..

[43] Cao X, Ma L, Gao B, Harris W (2009). Dairy-manure derived biochar effectively sorbs lead and atrazine. Environ. Sci. Technol..

[44] Zhang F (2015). Efficiency and mechanisms of Cd removal from aqueous solution by biochar derived from water hyacinth (Eichornia crassipes). J. Environ. Manag..

[45] Harvey OR, Herbert BE, Rhue RD, Kuo L-J (2011). Metal interactions at the biochar-water interface: energetics and structure-sorption relationships elucidated by flow adsorption microcalorimetry. Environ. Sci. Technol..

[46] Cui X (2016). Potential mechanisms of cadmium removal from aqueous solution by Canna indica derived biochar. Sci. Total Environ..

[47] Pignatello JJ, Mitch WA, Xu W (2017). Activity and reactivity of pyrogenic carbonaceous matter toward organic compounds. Environ. Sci. Technol..

[48] Xiao F, Pignatello JJ (2015). π+–π Interactions between (Hetero)aromatic amine cations and the graphitic surfaces of pyrogenic carbonaceous materials. Environ. Sci. Technol..

[49] Chang R, Sohi SP, Jing F, Liu Y, Chen J (2019). A comparative study on biochar properties and Cd adsorption behavior under effects of ageing processes of leaching, acidification and oxidation. Environ. Pollut..

[50] Liu H, Xu G, Li G (2021). Preparation of porous biochar based on pharmaceutical sludge activated by NaOH and its application in the adsorption of tetracycline. J. Colloid Interface Sci..

[51] Wongrod S (2018). Lead sorption by biochar produced from digestates: consequences of chemical modification and washing. J. Environ. Manag..

[52] Hall KR, Eagleton LC, Acrivos A, Vermeulen T (1966). Pore- and solid-diffusion kinetics in fixed-bed adsorption under constant-pattern conditions. Ind. Eng. Chem. Fund..

[53] Reguyal F, Sarmah AK, Gao W (2017). Synthesis of magnetic biochar from pine sawdust via oxidative hydrolysis of FeCl2 for the removal sulfamethoxazole from aqueous solution. J. Hazard. Mater..

[54] Lam YY, Lau SSS, Wong JWC (2019). Removal of Cd(II) from aqueous solutions using plant-derived biochar: kinetics, isotherm and characterization. Bioresour. Technol. Rep..

[55] Ding Z, Hu X, Wan Y, Wang S, Gao B (2016). Removal of lead, copper, cadmium, zinc, and nickel from aqueous solutions by alkali-modified biochar: batch and column tests. J. Ind. Eng. Chem..

[56] Tang J, Mu B, Zong L, Zheng M, Wang A (2017). Facile and green fabrication of magnetically recyclable carboxyl-functionalized attapulgite/carbon nanocomposites derived from spent bleaching earth for wastewater treatment. Chem. Eng. J..

[57] Li X, Xu J, Luo X, Shi J (2022). Efficient adsorption of dyes from aqueous solution using a novel functionalized magnetic biochar: Synthesis, kinetics, isotherms, adsorption mechanism, and reusability. Bioresour. Technol..

[58] Al-Rashdi B, Tizaoui C, Hilal N (2012). Copper removal from aqueous solutions using nano-scale diboron trioxide/titanium dioxide (B_2_O_3_/TiO_2_) adsorbent. Chem. Eng. J..

[59] Sun Q, Yang L (2003). The adsorption of basic dyes from aqueous solution on modified peat–resin particle. Water Res..

[60] Pholosi A, Naidoo EB, Ofomaja AE (2020). Intraparticle diffusion of Cr(VI) through biomass and magnetite coated biomass: a comparative kinetic and diffusion study. S. Afr. J. Chem. Eng..

[61] Hu X (2014). Effects of background electrolytes and ionic strength on enrichment of Cd(II) ions with magnetic graphene oxide–supported sulfanilic acid. J. Colloid Interface Sci..

[62] Moreno-Castilla C, Álvarez-Merino MA, López-Ramón MV, Rivera-Utrilla J (2004). Cadmium ion adsorption on different carbon adsorbents from aqueous solutions. Effect of surface chemistry, pore texture, ionic strength, and dissolved natural organic matter. Langmuir.

[63] Liu Z, Zhang F-S (2009). Removal of lead from water using biochars prepared from hydrothermal liquefaction of biomass. J. Hazard. Mater..

[64] Zhang Z (2019). A novel adsorbent of core-shell construction of chitosan-cellulose magnetic carbon foam: synthesis, characterization and application to remove copper in wastewater. Chem. Phys. Lett..

[65] Yousuf M (2016). Nypa fruticans as a potential low cost adsorbent to uptake heavy metals from industrial wastewater. Int. J. Appl. Bus. Econ. Res..

[66] Chai J-B (2020). Adsorption of heavy metal from industrial wastewater onto low-cost Malaysian kaolin clay–based adsorbent. Environ. Sci. Pollut. Res..

